# Necrotizing Enterocolitis: A Current Understanding and Challenges for the Future

**DOI:** 10.2174/0115733963318619240923062033

**Published:** 2024-09-26

**Authors:** Giuseppe De Bernardo, Carolina Vecchione, Carmen Langella, Carla Ziello, Grazia Parisi, Maurizio Giordano, Giuseppe Buonocore, Serafina Perrone

**Affiliations:** 1 Department of Woman and Child, Buon Consiglio Fatebenefratelli Hospital, Naples, Italy;; 2 Division of Pediatrics, Department of Translational Medical Sciences, University of Naples “Federico II”, Naples, Italy;; 3 Department of Clinical Medicine and Surgery, University of Naples “Federico II”, Naples, Italy;; 4 Department of Molecular and Developmental Medicine, University of Siena, Siena, Italy;; 5 Neonatology Unit, Department of Medicine and Surgery, University of Parma, Pietro Barilla Children's Hospital, Parma, Italy

**Keywords:** Preterm newborns, antibiotics, dysbiosis, low body weight, mother, metabolites, ultrasound, genetic predisposition

## Abstract

This perspective reviews the definition and current understanding of necrotizing enterocolitis and evaluates a future prevention approach to this multifactorial disease. An overview of the prevention approach in general is presented, where key aspects and emerging criticisms are identified. In addition, key elements of early diagnosis and treatment are presented, together with some of their challenges and ambiguities. Moreover, it concludes with emerging questions from the global community to reach a consensus on the definition, diagnosis, and management of necrotizing enterocolitis disease.

## 1 INTRODUCTION

Necrotizing enterocolitis (NEC) is a devastating gastrointestinal disease that occurs in 5-10% of preterm infants born under 1500 grams and has a mortality rate of 20-30% [1, 2]. The complex pathophysiology of NEC is not completely understood but culminates in an inflammatory response that can lead to irreversible intestinal injury [3]. The central pathogenic mechanism of NEC is thought to be exaggerated bacterial intestinal inflammation and necrosis, which in severe cases can lead to a systemic inflammatory response [3]. Risk factors for NEC include preterm delivery, in-utero growth restriction, antibiotic exposure, and absence of breast milk feeding [4, 5]. As NEC can develop suddenly, addressing risk factors that are potentially modifiable is a strategy to prevent NEC and improve patient outcomes [6]. This perspective aimed to critically examine the potential key strategy to prevent NEC and suggest some ways forward to achieve this objective [7-10].

## 2 RISK FACTORS OF NEC

In recent years, researchers have investigated a new set of tactics to reduce the potential risk factors for NEC [8]. Interesting results came from the modulation of Toll-like receptor signalling, exploration of genetic predispositions, the definition of metabolomic changes in NEC to discover potential biomarkers and of vascular endothelial growth factor (VEGF) signalling pathways, determination of relationships among IUGR, anaemia, and NEC [11, 12].

### 2.1 Genetic Predisposition

Some studies found an association between NEC and variants in regulators of the Toll-Like-Receptor 4 (TLR4) signalling pathway, NFκ-B1 variants, SNPs of IL-6 gene, NOD2 mutations, IL17F, and the allele of the autophagy (ATG) genes ATG16L1 [13]. TLR4, which is important for immunity against Gram-negative bacteria, has been implicated in NEC as studies reported elevated expression in intestinal samples obtained from NEC infants. Preliminary genetic studies on humans, however, have yet to yield strong evidence that genetic variations in the TLR pathway increase susceptibility to NEC. An important role is played by the single immunoglobulin interleukin-1-related receptor (SIGIRR), which modulates TLR signalling. Genetic variants resulting in loss of function of this receptor lead to an exaggerated inflammatory response to lipopolysaccharide exposure.

Autophagy plays an important role in inflammatory bowel disease and potentially also in NEC, which share many of the same pathways. The Sampath Laboratory recently discovered an association between variation in the autophagy gene, ATG16L1 (autophagy-related 16-like 1), and NEC in human neonates.

### 2.2 The Role of Antibiotics: Early and Prolonged Exposure Despite Sterile Haemoculture

Several studies have reported an increase in NEC incidence with increased duration of empirical early antibiotics and alteration of the gut microbiota [14]. Antibiotic administration is known to disrupt the composition of the gut microbiota, resulting in suppression of the beneficial bacteria Bifidobacterium and increased numbers of potentially pathogenic bacteria, such as Klebsiella, Enterobacter, Citrobacter, and Pseudomonas. Maternal antibiotic use in preterm or very low birth weight (VLBW) infants was found to be associated with a reduced incidence of NEC. It is possible that antibiotics given shortly before birth or intrapartum cause only transient changes in the microbiome, which are not associated with NEC in infants born VLBW or <32 weeks of gestational age. It supports the theory that prolonged empirical antibiotic treatment after birth is associated with an increased incidence of NEC and decreased infant safety. Based on these findings, prolonged antibiotic exposure may reduce the diversity of the gut microbiota, causing dysbiosis and predisposing preterm infants to NEC [12, 15-17].

### 2.3 Localised Intestinal Ischaemia-hypoxia-reperfusion Injury: Anaemia and IUGR

Several studies have demonstrated the co-existence of intestinal microcirculatory injury in children and NEC. Chronic fetal hypoxia due to extrinsic causes leads to the redistribution of blood flow to the vital organs and creates the risk of NEC. A combination of direct hypoxic-ischemic mucosal injury, susceptibility to stasis and prolonged ileus, and abnormal development of the gut microbiome with bacterial transmigration contribute to the increased incidence of NEC. Preterm infants with intrauterine growth restriction (IUGR) are a heterogeneous group and represent a significant proportion of neonatal intensive care unit (NICU) admissions. A subset of this IUGR population is identified antenatally as having abnormal umbilical artery Doppler flow velocities, predisposing them to NEC due to hypoxic mucosal injury [18]. Preterm infants with IUGR are twice as likely to develop NEC compared to term infants. The main cause of IUGR is placental insufficiency associated with increased oxidative stress in the placenta, which can be induced by pre-eclampsia and maternal hypertension [19]. The rearrangement of metabolic processes in the cell during hypoxia is regulated by hypoxia-induced transcription factors (HIFs). HIF promotes angiogenesis, which is regulated by VEGF and induces the expression of genes involved in the barrier function of the intestinal mucosa. Inappropriate timing of the reduction in HIF-mediated signalling may abolish VEGF production during a critical period in the development of the intestinal microvasculature, predisposing very preterm infants to NEC due to its underdevelopment [20]. Another important factor to consider is that preterm infants are at high risk of anaemia in early life [21]. Progressive anaemia is exacerbated by reduced blood flow to the gut due to the redistribution of blood flow to vital organs. The oxygen requirements of the intestinal mucosa, such as those associated with feeding, may not be met due to impaired oxygen delivery to the intestine. This may also contribute to the development of intestinal injury [22]. The possible mechanism explaining intestinal injury is that anaemia may reduce the expression of the tight junction protein ZO-1, increase the permeability of the intestinal barrier, and increase intestinal inflammation through altered macrophage function, leading to an intestinal injury that may increase the risk of NEC [21]. Blood transfusions are an integral part of treating anaemia and improving oxygen-carrying capacity in premature infants. Although these treatments are life-saving, in recent years, some clinical studies have linked red blood cell (RBC) transfusion to preterm or VLBW infants with an increased risk of NEC. Approximately 5.2-35% of premature infants receive a transfusion within 24-72 hours before the onset of NEC [21]. Transfusion-associated NEC (TANEC) has been associated with the occurrence of NEC (Bell's stage ≥ II or III) within 48 hours of RBC transfusion. Several mechanisms are thought to contribute to the development of TANEC, including impaired intestinal perfusion due to severe anaemia, exposure to immune triggers in transfused blood, upregulation of TLR4, activation of pro-inflammatory macrophages, and ischaemia-reperfusion injury [23].

### 2.4 Oxidative Stress

Preterm newborns are highly vulnerable to oxidative stress due to the high energy requirement for their growth and immaturity of antioxidant systems [24]. The imbalance between oxidant and antioxidant systems raises the level of free radicals that cause oxidative damage, especially in the gut. Preterm newborns affected by NEC showed higher levels of total antioxidant status, oxidative stress index, advanced oxidation protein products, total hydroperoxide, and non-protein-bound iron compared to control [25, 26].

## 3 NEC PREVENTION STRATEGIES

A strategy that has aroused interest in preventing NEC is the use of probiotics. Probiotics have a role in the optimization of the microbiome that is involved in NEC pathogenesis [27, 28]. Probiotics contribute to preventing disease in infants at risk of NEC by modulating gut [29]. European Society of Paediatric Hepatology, Gastroenterology, and Nutrition recommended the following probiotic strains to prevent NEC: L. rhamnosus GG, Bifidobacterium infantis BB-02, Bifidobacterium lactis BB-12, and Streptococcus thermophilus TH-4 [30]. Furthermore, it is important to consider the effects of prolonged use of antibiotics on gut microbiome, whereby they should be stopped promptly if blood cultures remain sterile for 24 to 48 hours [9]. A promising technique evaluated to prevent this disease is remote ischemic conditioning [31]. A delayed beginning of enteral feeding is considered a possible approach to reduce the incidence of NEC. Recent studies have reported that early trophic feeding with continuous daily increments compared to prolonging the day of initiation and slow/intermittent enteral feeding is associated with better body weight gain, shorter duration of parenteral nutrition, hospital stay, and reduced incidence of complications, such as NEC [32]. Breast milk represents the only factor consistently shown to reduce NEC due to some non-nutrient components of human milk that modulate gastrointestinal immune function and mucosal integrity, including IgA, hormones, polyunsaturated fatty acids, and oligosaccharides. The health benefits of human milk are well established, not only for premature infants but also for the prevention of other childhood diseases. When the mother's milk supply is insufficient, donor breast milk should be preferred to infant formula. Human milk banking is an absolute necessity, especially for preterm infants [33, 34].

The monitoring of oxygen saturation (SpO_2_) levels in the NICU is helpful to reduce hypoxia or hyperoxia exposure. The reference range of SpO_2_ is 90-95% in newborns <28 weeks of gestational age [35, 36]. Finally, early prevention and treatment of anaemia, strict evaluation of indications for transfusion, safe RBC product characteristics, and feeding protocols during transfusion may reduce the risk of NEC. Prospective studies with larger sample sizes are needed to better assess the influence of anaemia and transfusion on NEC [21].

## 4 KEY FEATURES OF THE EARLY DIAGNOSTIC MODALITIES

There are currently eight definitions of NEC, with similarities and differences in clinical signs and radiographic features. It is important to have a global consensus on the definition of NEC to improve management and outcomes. The first clinical staging of NEC was proposed by Dr Martin Bell in 1978 and successively by Patel *et al.* in 2020 (Table **1**) [7].

Nowadays, it remains the most widely used method for the diagnosis of NEC, despite its potential limitations: contamination by spontaneous intestinal perforation (SIP), high incidence of stage I, uncertainty about the presence of pneumatosis, lack of consideration of baseline risk (*e.g.*, gestational age), and lack of explicit case definition. NEC diagnosis can also be obtained at surgery or on postmortem examination based on radiologic and clinical criteria, according to the Vermont Oxford Network criteria [7]. The prevailing standard imaging method for diagnosis and follow-up of NEC is abdominal radiography, but early signs of NEC are neither sensitive nor specific. Ultrasound should be considered an adjunct to abdominal radiography as it provides direct, real-time visualization and assesses bowel wall thickening/thinning, echogenicity, perfusion, and peristalsis. It has the potential to appropriately stage NEC by detecting pneumatosis, portal venous gas, free gas, fluid and fluid quality, and Doppler signals in mesenteric vessels. Furthermore, it does not involve radiation, is widely available, is easy to perform after training, and should be promoted in a multidisciplinary approach [10]. Regarding the treatment of NEC, the non-perioperative (NPO) strategy is used in cases of mild or suspected NEC, even if it is not clear about the correct duration of NPO. The classical antibiotic regimen (ampicillin, gentamicin, and metronidazole or clindamycin for 10-14 days) is used in cases of moderate clinical stage, but the safety and efficacy of the latter two drugs in the paediatric population are unknown, as there are no prospective or randomized studies on them.

New biological molecules may be used to treat NEC. Heparin-conjugated epidermal growth factor-like growth can reverse NEC in *in-vivo* animal experiments by stimulating mucosal healing. C34, a 2-acetamidopyranoside, has been utilized to attenuate the inflammation of the gut by inhibition of TLR4 signaling. Breast milk oligosaccharides and growth factors contained in amniotic fluid can improve NEC treatment, reducing the inflammation status.

Additionally, orally administered lactoferrin is effective in blocking the development of NEC. However, further randomized clinical trials are needed to better understand the clinical application of these new treatments [8]. Surgical treatment is necessary for children with severe symptoms. However, there is no standardization of the criteria used to decide surgery. There is also controversy about the timing of surgery, the method of surgery, and the management of the post-operative period [7, 8].

## 5 PATIENT-CENTERED PERSPECTIVE

There is an urgent need to understand the long-term complications of NEC to improve counseling on prognosis and treatment among surviving patients, parents, and healthcare providers. In 2023, Canvasser *et al.* revealed that nearly three-fourths of the parents showed increased anxiety, worry about the future, post-traumatic stress disorder, and difficulty sleeping. Further research could quantify the costs of long-term NEC complications and improve the availability of resources to face the financial burdens of illness for survivors and families [37].

## CONCLUSION

Researchers still seek to identify potential risk factors for NEC development, study genetic, environmental, and bacteriological elements, and allow for individualized clinical management. For a better clinical prognosis of NEC, research will improve the diagnostic modalities of this disease, including the identification of biomarkers and more accurate imaging techniques, as described above. It is hoped that the early diagnosis of NEC can lead to prompt intervention to potentially diminish the severity of the disease and minimize the need for operative intervention. Additional emphasis is now being placed on NEC prevention, including the practical use of donor breast milk, probiotics, and the development of an adaptive formula that is endowed with the benefits of breast milk.

## AUTHORS’ CONTRIBUTIONS

BG contributed to the conception and design of the study. GM, BG, and PS carried out the analysis and interpretation of the results. VC, LC, ZC, and PG drafted the manuscript. All authors reviewed the results and approved the final version of the manuscript.

## Figures and Tables

**Table 1:** Comparison of intestinal signs and radiologic findings across NEC definitions.

**Variable Category**	**Bell Staging**			**Modified Bell Staging**						**UK**	**VON**	**CDC**	**2 of 3**	**ST**	**INC**
	**I**	**II**	**III**	**IA**	**IB**	**IIA**	**IIB**	**IIIA**	**IIIB**						
**Intestinal signs**	-	-	-	-	-	-	-	-	-	-	-	-	-	-	-
Poor feeding	+	+	+	-	-	-	-	-	-	-	-	-	-	+	-
Emesis	+	+	+	+	+	+	+	+	+	-	+	+	-	-	-
Pre-gavage residuals	+	+	+	+	+	+	+	+	+	+	-	-	-	-	-
Bilous aspirates	+	+	+	-	-	-	-	-	-	+	+	+	-	-	-
Abdominal distention (mild)	+	+	+	+	+	+	+	+	+	+	+	+	+	-	+
Marked distention	-	+	+	-	-	-	-	+	+	-	-	-	-	-	-
Guaiac-positive stool	+	+	+	+	+	+	+	+	+	-	-	-	-	-	-
Rectal bleeding (occult)	+	+	+	-	+	+	+	+	+	+	+	+	+	-	+
Marked haemorrhage	-	-	+	-	-	-	-	-	-	-	-	-	-	-	-
Absent bowel sounds	-	-	-	-	-	+	+	+	+	-	-	-	-	-	-
Abdominal tenderness	-	-	-	-	-	+	+	+	+	+	-	-	-	-	-
Marked tenderness	-	-	-	-	-	-	-	+	+	-	-	-	-	-	-
Generalized peritonitis	-	-	-	-	-	-	-	+	+	-	-	-	-	-	-
Abdominal cellulitis	-	-	-	-	-	-	±	+	+	-	-	-	-	-	-
Right low quadrant mass	-	-	-	-	-	-	+	+	+	-	-	-	-	-	-
Abdominal discoloration	-	-	-	-	-	-	-	-	-	+	-	-	-	+	-
**Radiologic findings**	-	-	-	-	-	-	-	-	-	-	-	-	-	-	-
Normal	-	-	-	+	+	-	-	-	-	-	-	-	-	-	-
Ileus	+	+	+	+	+	+	+	+	+	-	-	-	-	-	-
Pneumatosis	-	+	+	-	-	+	+	+	+	+	+	+	+	+	+
Portal venous gas	-	+	+	-	-	-	+	+	+	+	+	+	+	+	+
Ascites	-	-	-	-	-	-	+	+	+	-	-	-	-	-	-
Pneumoperitoneum	-	-	+	-	-	-	-	-	+	+	+	+	-	-	-
Fixed loop	-	+	+	-	-	-	-	-	-	+	-	-	-	-	-
Small bowel separation	-	+	+	-	-	-	-	-	-	-	-	-	-	-	-

**Note: ** + = Condition present; **Abbreviations:** UK=United Kingdom; VON= Vermont Oxford Network; CDC= Centers for disease control and prevention; ST= Stanford; INC= International neonatal consortium.

## LIST OF ABBREVIATIONS

ATG: Allele of the Autophagy
HIF: Factors Induced by Hypoxia
IUGR: Intrauterine Growth Restriction
NEC: Necrotizing Enterocolitis
NICU: Neonatal Intensive Care Units
NPO: Non-per-os.
RBC: Red Blood Cell
RIC: Remote Ischemic Conditioning
SIGIRR: Single Immunoglobulin Interleukin-1-related Receptor
SIGNEC: Special Interest Group in Necrotizing Enterocolitis
SIP: Spontaneous Intestinal Perforation
SpO_2_: Oxygen Saturation
TANEC: Transfusion-associated NEC
TLR: Toll-like Receptors
VEGF: Vascular Endothelial Growth Factor
VLBW: Very Low Birth Weight
